# Dietary Grape Pomace Supplementation in Lambs Affects the Meat Fatty Acid Composition, Volatile Profiles and Oxidative Stability

**DOI:** 10.3390/foods12061257

**Published:** 2023-03-16

**Authors:** Francesca Bennato, Camillo Martino, Andrea Ianni, Claudia Giannone, Giuseppe Martino

**Affiliations:** 1 Department of BioScience and Technology for Food, Agriculture and Environment, University of Teramo, 64100 Teramo, Italy; 2 Department of Veterinary Medicine, University of Perugia, 06126 Perugia, Italy

**Keywords:** grape pomace, lamb meat, rumenic acid, hexanal

## Abstract

The aim of this study was to evaluate the effects of supplementing grape pomace (GP) in lambs’ diets. A total of 30 lambs homogeneous for body weight (13.1 ± 2.1 kg) and age (25–30 days) were randomly allocated into two groups. The control group (CTR) received a standard diet for 45 days, while in the same period the experimental group (GP+) was fed with a diet containing 10% GP on a dry matter (DM) basis. The meat samples from the two groups showed no significant differences in drip loss, cooking loss, meat color and total lipid amount. However, the experimental feeding strategy influenced the meat fatty acid composition, with an increase in the relative percentages of stearic, vaccenic and rumenic acids. In particular, the increase in rumenic acids is associated with several health benefits attributed to its high bioactive properties. In cooked meat samples stored for 5 days at 4 °C, the dietary GP supplementation induced an increase in nonanal and 1-octen-3-ol and a significant reduction of hexanal, an indicator of oxidation; this improved resistance to oxidation in the GP+ samples and was also confirmed by the thiobarbituric acid reactive species (TBARS) test. In summary, the present study showed that the dietary GP supplementation was effective in improving the fatty acid composition and the oxidative stability of lamb meat. The use and valorization of the GP as a matrix of interest for zootechnical nutrition can, therefore, represent a suitable strategy for improving the qualitative aspects of animal production.

## 1. Introduction

Grape pomace (GP) is the major by-product of the wine industry. Specifically, this waste comes from the grape (*Vitis Vinifera*) pressing process, which obtains wine, and constitutes skins, seeds and stems. Approximately, 20% of the weight of processed grapes remains as GP and is produced by the wine industry in millions of tons; its disposal is associated with ecological and economic issues [1]. In this regard, it is notable that based on the data from the International Organization of Vine and Wine (OIV), of the ten major red wine producing countries, Italy is the highest contributor of GP. The GP matrix is particularly rich in phenolic compounds with a high antioxidant capacity, where the most abundant compounds are antochyanins, hydroxybenzoic and hydroxycinnamic acids, flavan-3-ols, flavonols and stilbenes [2].

The significant biological properties of these families of compounds have directed considerable interest to this by-product, justifying the attempts in recent years to use GP as an ingredient in farm animal nutrition. With specific regard to meat quality, GP has shown several positive effects when included in the animal’s diet. By way of example, the study conducted by Martin-Flores et al. [3] investigated the use of GP as a dietary supplement for lambs. The GP addition to the lamb’s diet resulted in an increase in total lipids, higher levels of polyunsaturated fatty acids (PUFA) and a greater oxidative stability. In particular, the inhibition of the last steps of rumen biohydrogenation (BH), with a consequent increase in the concentration of linoleic acid (C18:2 n6), was observed. Research conducted by Gomez-Cortes et al. [4] studied the effects of seed or grape residues at levels up to 10% in ewes’ diets and found a higher accumulation of vaccenic acid. Kafantaris et al. [5] investigated the effects of GP supplementation on the meat quality from lambs. The inclusion of GP in lambs’ diets improved their performance during the experimental trial. Furthermore, the dietary GP supplementation led to significantly higher contents of arachidic (20:0), eicosadienoic (20:2 *n* − 6 cis-11, 14), eicosapentaenoic (20:5 *n* − 3) and docosahexaenoic (22:6 *n* − 3) acids. In this regard, winemaking by-products have been associated with an improved oxidative stability also in beef, with a concomitant increase in linoleic acid concentration in meat and a consequent increase in the PUFA: saturated fatty acids (SFA) ratio, which is typically low in ruminants [6]. This highlighted a positive aspect considering that the meat from ruminants is characterized by high concentrations of SFA that may constitute a public health concern, due to their association with cardiovascular diseases [7].

Studies exploring the use of GP in lamb feeding have mainly focused their attention on dry matter digestibility, growth performance and carcass and meat quality traits. In this context, less attention has been given to the meat oxidative stability. When this topic was addressed, reference was made to the use of grape seed extracts in animal feeding [8]; therefore, a different matrix from the one utilized in this study, or the antioxidant potential was evaluated through the analysis of enzymes associated with the antioxidant response, such as glutathione peroxidase, superoxide dismutase and catalase [9]. To our knowledge there is less information regarding the monitoring of the oxidative stability through the characterization of the volatile profiles following the cooking of the meat. This approach makes it possible to obtain information not only on the potential accumulation of compounds capable of altering the product flavor and taste, but also in the formation of elements closely associated with the onset and progression of oxidative events, mainly involving the lipid component. Our hypothesis is that the dietary GP should preserve the oxidative stability of the product, limiting the lipolytic processes and thus avoiding the accumulation of volatile organic compounds (VOCs), especially aldehydes, which are harmful to the meat organoleptic properties, as well as to the consumers’ health.

## 2. Materials and Methods

All animal procedures were approved by the national legislation on animal welfare (DL n. 146, 26 March 2001, EC Directive 58/98/EC). Animals were slaughtered in accordance with Regulation 1099/2009 of the European Union on the protection of animals at the time of killing. The scope of this investigation involved all the activities being conducted on a commercial farm and animals were not exposed to breeding practices outside of those commonly used; as a result, no further ethical considerations were deemed necessary.

### 2.1. The Experimental Design and Sample Collection

The study was conducted between March and April (5–14 °C temperature and 34–72% relative humidity) in a farm located in central Italy (Teramo, TE, Abruzzo). A total of 30 lambs with similar body weights (13.1 ± 2.1 kg) and ages (25–30 days) were randomly and equally assigned to two groups (15 animals per group) and allotted two separate holding pens. The lambs were individually fed twice a day, the control group (CTR) received a standard diet, formulated based on the National Research Council’s [10] recommendation of a pelleted mixed diet, in which the main ingredients were: maize, fine bran, barley meal, soybean meal, sunflower oil, vitamins and mineral premix. The experimental group (GP+) was fed with the same diet, but containing 10% GP on a DM basis. The GP used in the trial was derived from red wine grape pomace (*Vitis vinifera* L.) and was prepared as previously described [6]. Briefly, red wine grape pomace obtained from a winery was dried and milled. Subsequently, the by-product was treated with water at 90 °C, to recuperate the tartaric acid, and dried in order to better preserve it. The content of total phenolic compounds (TPCs), the antioxidant activity (AOA) and the fatty acid profiles of the GP and the standard diets were also investigated according to the protocols reported by Bennato et al. [11].

At the end of the trial, all lambs, aged 73–78 days and weighing 20.50 ± 1.60 kg and 21.30 ± 1.80 kg, respectively, for CTR and GP+, were fasted for 12 h and given unrestricted access to water before slaughter in a commercial abattoir. Carcasses were left for 48 h at 4 °C in a cold room and covered by a synthetic film in order to avoid contact between the carcass and the surrounding environment. After 48 h, the *longissimus dorsi* muscle was removed from the left side of all the carcasses. Samples of approximately 100 g were extracted and immediately stored at −20 °C for the fatty acid profile determination. Conversely, the evaluations concerning drip loss (DL) and cooking loss (CL) were performed immediately on raw meat after the sampling of the *longissimus dorsi* (T2), while color was evaluated also on raw meat storage at 4 °C until 7 (T7) days *post mortem*. Additionally, the effect of cooking on the accumulation of VOCs was evaluated. In particular, the evaluation was performed on the same samples used for cooking loss, which were frozen both after cooking (T0) and after 5 days (T5) of cooked sample storage at 4 °C. These samples were also analyzed by the TBARS-test in order to evaluate in more detail the extent of the oxidative processes eventually induced by cooking and subsequent storage.

### 2.2. Evaluation of Meat Color

Color evaluations were performed as previously reported [6] on the transverse section of meat samples (2–2.5 cm thickness) after 30 min of blooming at 4 °C, using the Minolta-CR 300 (Minolta Co, Osaka, Japan), with a D65 illuminant, a 10° standard observer angle and a 30 mm aperture. All analyses were performed on 3 different areas of each sample, based on the CIELAB system (L* = lightness, a* = redness, b* = yellowness). Before starting, the instrument was calibrated with a white tile (L* = 100) and a black glass (L* = 0). The evaluation was performed on both T2 and T7 raw meat samples in order to highlight variations induced by storage at 4 °C.

### 2.3. Drip Loss, Cooking Loss, and the Chemical Composition of Meat Samples

The DL analysis was carried out to determine the propensity of raw meat to retain water according to the method of Honikel [12]. Meat samples of 2–2.5 cm thick weighing 80 g were suspended in an inflated plastic bag and stored at 4 °C for 24 h. After this period, the samples were re-weighed after carefully and gently drying the meat surface with absorbent paper. The DL was calculated according to the following formula:

(1) $$DL\left(\%\right)=\frac{\left(W_{0}-W_{f}\right)}{W_{0}}\times 100$$

where W_0_ is the initial sample weight expressed in g and W*_f_* is the final sample weight expressed in g.

The CL was calculated as reported above Equation (1) and expressed as a percentage of weight loss after cooking according to the procedure of Honikel [12], with slight modifications. Briefly, 2–2.5 cm thick samples weighing 80 g were placed in plastic bags and cooked in a water bath (Grant Instruments Ltd., Barrington, UK) until reaching an internal temperature of about 75 °C. The samples were left to cool at room temperature and kept overnight at 4 °C before weighing.

The meat moisture (AOAC 950.446) and fat (AOAC 985.15) content were determined according to the official methods of AOAC [13].

### 2.4. Fatty Acid Profiles of Meat and Lipid Oxidation

The total lipids were extracted with the Folch method [14]. Five grams of meat was minced using an Ultra-Turrax in 90 mL of Folch solution (chloroform: methanol, 2:1). Subsequently, samples were kept under constant agitation for 6 h at room temperature and then transferred into separating funnels with the addition of 30 mL of 1% NaCl; the chloroform phase was evaporated to dryness with a Strike-Rotating Evaporator (Steroglass S.r.l., Perugia, Italy) set at 38 °C. For each sample, the formation of the fatty acid methyl esters (FAMEs) was induced by mixing 70 mg of fat with 1 mL of hexane and 500 µL of sodium methoxide. The FAME detection was conducted by a gas chromatograph (Focus GC; Thermo Scientific, Waltham, MA, USA) equipped with a capillary column (Restek Rt-2560 Column fused silica 100 m × 0.25 mm highly polar phase; Restek Corporation, Bellefonte, PA, USA) and a flame ionization detector (FID). Hydrogen was used as the carrier gas. ChromeCard software (Version 2.12, Thermo Fisher Scientific, Milan, Italy) was used to calculate the peak areas and the value of each FA was reported as a relative percentage of the total FAMEs. The identification of individual FAMEs was performed by comparing the retention time of the standard mixture FIM-FAME7-Mix and C18:1 trans-11 (Matreya LLC, State College, PA, USA). The values associated with each FA were used to calculate the sum of SFA, MUFA and PUFA; furthermore, the atherogenic (AI) and thrombogenic indices (TI) were also calculated, as previously described [15].

### 2.5. Analysis of Volatile Compounds

The VOCs estimation was performed with a gas chromatograph (Clarus 580; Perkin Elmer, Waltham, MA, USA) coupled with a mass spectrometer (SQ8S; diameter: 30 × 0.25 mm; film thickness: 0.25 µm; Perkin Elmer). In brief, 10 mL of an aqueous solution of saturated NaCl (360 g/L) and 10 µL of internal standard (3-methyl-2-heptanone; 10 µg/L in ethanol) were mixed with 5 g of minced cooked meat and exposed to a divinylbenzene-carboxen-polydimethylsiloxane SMPE fiber (length: 1 cm; film thickness: 50/30 µm; Sigma-Aldrich, Milan, Italy) at 40 °C for 1 h. After adsorption time, the extracted VOCs were thermally desorbed into the gas chromatograph injector in splitless mode for 30 min at 250 °C. The oven temperature was held at 50°C for 1 min, increased at a rate of 3 °C/min up to 200 °C and held for 1 min, and then increased from 200 °C to 250 °C at 15 °C/min and held for 15 min. Helium was used as a carrier gas at a flow rate of 1 mL/min. Following this, the extracted VOCs were identified as previously described [6].

### 2.6. Lipid Oxidation

Lipid oxidation was evaluated with the TBARS-test according to the procedure reported by Grotta et al. [16]. To sum up, 500 µL of 0.1% butylated hydroxytoluene in methanol were combined with 3.5 g of frozen meat to stop oxidation. Subsequently, the Ultra Turrax T25 was used to homogenize the samples with 50 mL of an aqueous solution containing 7 % trichloroacetic acid, before distillation, and an equal volume of a solution containing 0.02 M thiobarbituric acid in 90% acetic acid added to each distillate. This solution was kept for 1 h in a thermostated bath at 80 °C and after cooling, the absorbance at 534 nm of this preparation was assessed. The amount of MDA was calculated using a calibration curve (R^2^ = 0.9897) and the results were expressed in mg of MDA per kg of meat (ppm).

### 2.7. Statistical Analysis

All the analyses were conducted in triplicate and the results were reported as mean ± standard deviation (SD). The data were collected using Microsoft Office Excel 2021 (Version 2108, Washington, USA), and performed using the Statistical Analysis System (SAS) package (SAS Institute, Cary, NC, USA). Student’s t-test was applied in order to identify significant differences between the two groups in relation to the impact of the feeding treatment. Color, volatile profiles and oxidative stability were processed with an Anova (Analysis of Variance) to analyze the impact of the feeding treatment, and the time of storage, on meat quality. Mean values were assessed by HSD Tukey’s test and the statistical significance was declared for *p* values lower than 0.05 and 0.01.

## 3. Results

### 3.1. Fatty Acid Profiles, Polyphenol Content and Antioxidant Activity in the GP and Standard Diets

As reported in Table 1, linoleic acid (C18:2 c9, c12) was the most abundant fatty acid in the GP, followed by oleic (C18:1 c9) and palmitic (C16:0). Stearic (C18:0) and linolenic (C18:3 c9, c12, c15) acids were less represented. A comparison of the two diets showed no significant differences; however, the *p* value calculated for linoleic acid (C18:2 c9, c12) was very close to significance (*p* = 0.061). The whole GP extract showed a high content of TPCs (73.36 ± 7.66 mg GAE g^−^^1^ DM) and the AOA was 496.12 ± 19.23. Significant differences in TPCs and the AOA were found between the two diets (*p* < 0.05).

### 3.2. The Physical and Chemical Composition of the Meat

As shown in Table 2, the dietary GP supplementation did not significantly alter DL and CL. Similarly, no significant variations were found for meat moisture and total lipid content (*p* > 0.05).

With regard to the meat color (Table 3), the considered chromatic coordinates (L*, a* and b*) did not undergo significant variations between the experimental treatments (*p* > 0.05) at either T2 or T7. On the other hand, significant changes seem to have been induced by the storage time, with the T7 samples showing lower redness values (*p* < 0.01).

### 3.3. Fatty Acid Profiles

The fatty acid composition of lamb meat is reported in Table 4. The evaluation of the fatty acid profiles showed a significant increase in the concentration of stearic acid (C18:0; *p* < 0.05), due to the dietary supplementation with GP. No significant differences in MUFA and PUFA were observed between the two groups; however, in GP+ samples vaccenic (C18:1 t11; *p* < 0.01) and rumenic (CLA; *p* < 0.01) acids increased significantly. In addition to rumenic acid, the only identified PUFA were linoleic acid (C18:2 c9, c12) and linolenic acid (C18:3 c9, c12, c15), which did not undergo variations as a consequence of dietary supplementation. No significant differences in TI and AI were found between the two groups.

### 3.4. Identification of Volatile Compounds in Meat Samples

The characterization of the volatile profiles in cooked meat samples led to the identification of 16 compounds (both at T0 and T5), belonging to the family of aldehydes, alcohols, ketones and phenolic compounds (as shown in Table 5). Significant differences between GP+ and CTR were found only in the T5 meat samples. In both groups, independent of the storage time, the most represented compound was hexanal, whose relative percentage increased in both GP+ and CTR after 5 days of storage. However, in T5 GP+ samples, the hexanal was significantly lower compared to the CTR (*p* < 0.05). The storage time induced a decrease in 1- Pentanol and 1-Octanol and an increase in 2-Octen-3-ol and 2_Octen-1-ol. With specific regard to the aldehydes detected in T5 samples, the GP intake also induced an increase in the concentration of nonanal (*p* < 0.05). Among the alcohols, 1-Octen-3-ol was observed to be higher (*p* < 0.01) in T5 samples obtained from lambs fed the dietary supplementation. In both groups, the storage time induced a decrease in 1-Pentanol and 1-Octanol and an increase in 2-Octen-3-ol. Regarding dietary effect, no significant differences were observed among the aromatic hydrocarbons, ketones and esters. On the contrary, the storage time induced a decrease in the relative percentage of 2,3-Octanedione.

### 3.5. Oxidative Stability of Cooked Meat

**Figure 1:**
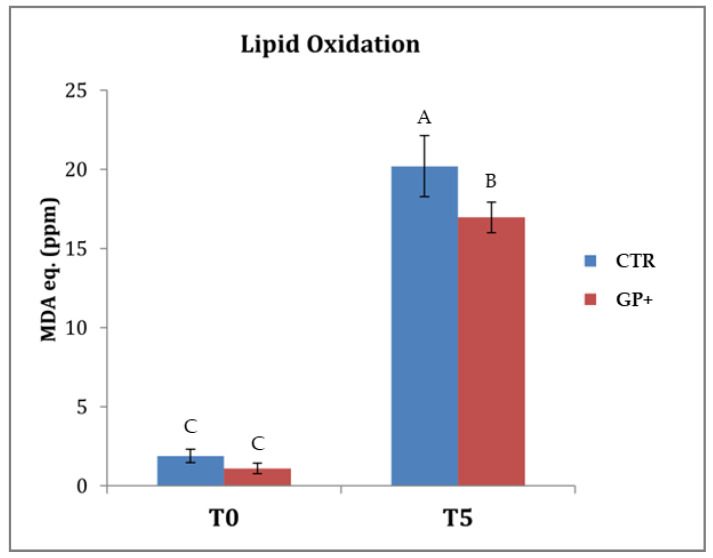
The results of the thiobarbituric acid reactive species test (TBARS-test) on cooked meat samples obtained from lambs fed with a standard diet (CTR) and lambs fed a dietary supplementation with grape pomace (GP+). The analysis was performed after cooking (T0) and after 5 days of storage (T5) at 4 °C. ^A,B,C^ Different letters indicate significant differences due both to the storage time and to the feeding treatment (*p* < 0.05).

The oxidative stability of the cooked meat was evaluated with the TBARS-test. As shown in Figure 1, immediately after the heat treatment no significant variations were evidenced between samples belonging to the two experimental groups. As expected, during the storage a significant increase in MDA was noted in both groups. However, after 5 days of storage of the cooked samples at 4 °C, the GP+ meat was characterized by a lower oxidative process than that for the CTR samples (*p* < 0.05).

## 4. Discussion

The aim of this study was to evaluate the effect of a dietary supplementation with GP on lamb meat quality, with particular attention to the fatty acid composition, volatile profiles and oxidative stability.

A key factor in determining the quality of meat is the water holding capacity (WHC), which affects the meat appearance, color, tenderness and juiciness after cooking [17]. In order to estimate the muscle ability to retain the water content there are two recognized criteria: cooking loss and drip loss. In our study no significant differences in drip loss and cooking loss were found between the two experimental groups. Similarly, to the present study, Flores et al. [18] found no change in cooking loss in the meat of lambs fed diets containing different levels of GP (0%, 25%, 37.5% and 50%), which replaced whole corn silage with a ratio of almost 50:50 between forages and concentrates. It is well known that dietary antioxidants have been shown to have a number of positive effects on drip loss. This is likely because these substances have the potential to maintain the integrity of the mitochondrial membranes by inhibiting the activity of phospholipase A_2_ [19], which consequently lowers the release of Ca^++^ and the liberation of long-chain phospholipids from the mitochondria, reducing post-mortem glycolysis and drip loss [20]. However, none of the antioxidants presumably contained in GP had an effect on the cooking loss in this study.

With regard to animals of zootechnical interest, this mechanism seems to have been better characterized in monogastrics; specifically, the role of the phospholipase A_2_ activity in normal and PSE pork has been clearly described [21]. Furthermore, the ability of the dietary grape phenolic compounds to interfere with the enzyme activity has been amply demonstrated in trials conducted on murine models [22], in which the simultaneous reduction of inflammation mediators was highlighted, with a significant improvement in animal health. The lack of a significant difference in drip loss could depend on the fact that the dose of GP administered to the animals in our trial is not enough to determine an adequate availability at mitochondrial level of the phenol compounds capable of interfering with the A_2_ phospholipase activity. In this regard, it should be reported that in other studies the dietary supplementation has been brought up to 20% of dry substance [23].

Furthermore, the chemical composition of meat samples was not influenced by the dietary treatment, confirming what has been previously discussed by Massaro Junior et al. [24] who showed that the addition of 0, 10, 20 and 30% of GP silage to the diets of lambs did not affect the meat quality attributes, such as lipids and moisture.

Color is an important component of food quality, both in raw and cooked meat. This is especially true because color significantly influences how consumers perceive quality and affects how they consider other essential aspects like flavor and aroma. In the present investigation, no statistically significant differences in color due to the dietary treatment were observed between the two groups. Since brightness (L*) and moisture content are frequently connected in food matrices of different sources, this finding can be at least partially explained by the lack of significant changes in moisture content [25]. Despite this consideration, the finding associated with L* in this study remains quite unexpected. In fact, the GP coming from red grapes should be rich in antocyanins that, in addition to being characterized by their high therapeutic potential due to their antiradical and antioxidant action, represent powerful natural colorants able to induce a dark coloration if present in food matrices [26]. In agreement with our results, Zhao et al. [9] showed similar findings regardless of diet, attributing the lack of color variation in lamb meat, compared to other species like pork, to an overall darker pigmentation that obscures any color enhancement. With specific regard to the chromatic coordinate a* that is associated with redness, the first consideration that can be advanced is that in both experimental groups, the values obtained for T0 raw meat are far above the threshold value of 9.5, which consumers perceive to be an acceptable lamb meat color [27]. The redness value did not significantly differ between the two groups, but significantly decreased as an effect of the storage time. There are no similar studies on lamb meat which can be used to discuss this evidence in depth; however, it could be interesting to refer to Garrido et al. [28] who conducted a trial which examined the effects of red GP extracts on the meat quality of pork burgers. These authors discovered that the addition of GP resulted in color stability after meat storage, an aspect that was associated with lowering lipid oxidation, probably as an effect of the strong antioxidant action of the GP extracts used. Specifically, these findings assumed that lipid oxidation and myoglobin degradation are closely related; therefore, the oxidation products, such as aldehydes, could actively promote the color degradation [29].

The dietary supplementation positively affected the fatty acids composition in the lamb meat, a finding of great interest if we consider the health value associated with the fatty acid profiles in foods [7]. The most interesting aspect of the present experiment is certainly associated with the presence of higher relative percentages of trans-vaccenic acid and CLAs in the GP+ samples. Most CLAs are produced by ruminants endogenously, through an enzymatic mechanism that uses the trans-vaccenic acid as a substrate [30]. The trans-vaccenic acid, in turn, accumulates in the rumen as a result of the biohydrogenation process, which tends to remove the unsaturations at the level of the PUFA consumed in the animal’s diet. This phenomenon preferentially involves linoleic acid, which showed higher concentrations in the experimental diet with a difference between the two groups close to significance (*p* = 0.061). It is therefore plausible that the diet has favored an increase in the synthesis of trans-vaccenic acid, making a higher quantity of substrate available to the endogenous pathway of CLA synthesis. This greater accumulation of CLA is of considerable interest due to the high health value associated with these compounds. This value is attributed to an important antioxidant activity able to limit the accumulation of the reactive oxygen species and protect the ruminants’ tissues from lipoperoxidation [31]. Several studies also evidenced the different health benefits induced by CLA for humans; specifically, these compounds modulated the immune system, to reduce the body fat accumulation, improve the bone mineralization and slow down the atherosclerosis development [32,33,34,35].

With regard to the meat fatty acid profiles, similar findings were also highlighted in other trials that involved the use of GP in the nutrition of animals of zootechnical interest, both ruminants and monogastrics [6]. However, it is also useful to report observations by Kafantaris et al. [5], who highlighted that the integration of GP into lambs’ diets induced an increase in the content of long chain n-3 fatty acids, reducing the n-6/n-3 ratio with respect to the control group. This discrepancy could depend, initially, on the fact that in this trial animals with different genetics (Chios breed) were used, as well as the experimental diet having involved silage with polyphenolic additives from GP and not dried GP as in our study. Moreover, it should be emphasized that the evaluations were carried out on quadriceps muscles, a different anatomical region from the *longissimus dorsi*, which was sampled in our study.

Different studies showed how variations in the diets of animals of zootechnical interest induced variations in the accumulation of volatile compounds in meat [6]. Most of these compounds, capable of influencing the aroma and taste of the product, tend to accumulate following cooking or heat treatments, which trigger a series of reactions. Specifically, these are multi-directional reactions that originate under the influence of temperature from non-volatile substrates that are present in raw meat. The volatile compounds are mainly released following Maillard reactions, lipid oxidation or interactions between the products of the Maillard reaction with the oxidation products [36].

In the present study, we investigated the VOCs composition from the *longissimus dorsi* muscle. Following cooking, the majority of these compounds were aldehydes, alcohols, aromatic hydrocarbons, ketones and esters. The most represented was hexanal, which was significantly lower in the GP+ samples after 5 days of meat storage at 4 °C. The accumulation of this compound in meat is generally correlated to the onset of oxidative events affecting the lipid component. As evidenced in other studies, this aldehyde preferentially derives from the oxidation of linoleic acid (C18:2 c9, c12) in muscle [37]. Since the experimental group (GP+) had a higher percentage of linoleic acid, which was nearly significant, this could represent a contradiction. However, as advanced in other studies [6], our hypothesis is that the bioactive substances in the grape pomace, presumably phenolic in nature, may have slowed down the oxidative processes performing an antioxidant activity. In addition, hexanal confers a herbal and rancid taste [37,38]; therefore, as well as being a reliable indicator of oxidation, its presence is also associated with deleterious aspects from a sensorial point of view. Another interesting difference induced by the dietary supplementation with grape pomace is the presence of higher concentrations of 1-octen-3-ol following the storage period of the cooked meat. This VOCs has been identified in different types of meat [39,40] as well as in mushrooms [41], and its insecticidal and antimicrobial activity has been characterized [41,42]. The presence of this compound is, therefore, associated with positive aspects as regards the shelf-life and safety of the food product. However, as previously reported by Mariutti et al. [43], alcohols like 1-octen-3-ol, which are produced by the oxidation of linoleic and arachidonic acids, have lower threshold values and a rusty, mushroom-like stench that might not find approval from some consumers. For that reason, the question regarding its contribution to the meat organoleptic profile deserves more accurate and targeted evaluations.

## 5. Conclusions

In this study, it was shown that adding grape pomace to the diet of lambs led to a significant rise in the levels of CLA in meat. This family of fatty acids is usually associated with a higher health functionality, although it is difficult to determine whether the observed increases could be responsible for significant and appreciable nutraceutical effects.

Based on the current findings, grape pomace may be used as an ingredient in small ruminant nutrition as a sustainable way for improving specific qualitative aspects of meat, focusing on the increase in the oxidative stability, which is directly related to the shelf-life of the product and, therefore, to its permanence on the market.

## Figures and Tables

**Table 1:** Fatty acid profiles, total phenolic content and antioxidant activity of grape pomace (GP) and the custom-formulated diet for the control group (CTR) and experimental group (GP+).

	GP	CTR	GP+
Fatty acids ^1^ (%)			
C16:0	11.31 ± 1.26	19.64 ± 0.40	18.94 ± 2.37
C18:0	3.90 ± 0.80	3.34 ± 0.06	3.74 ± 0.35
C18:1 c9	15.10 ± 2.87	22.87 ± 0.08	22.48 ± 0.57
C18:2 c9, c12	66.75 ± 4.05	51.62 ± 0.51	52.36 ± 1.46
C18:3 c9, c12, c15	2.79 ± 2.06	2.50 ± 0.02	2.44 ± 0.04
SFA	15.21 ± 0.87	22.99 ± 0.47	23.68 ± 2.02
MUFA	15.10 ± 0.23	22.87 ± 0.08	22.48 ± 0.57
PUFA	69.54 ± 0.68	54.12 ± 0.53	53.81 ± 1.43
DM (%)	33.95 ± 1.50	89.00 ± 0.20	88.00 ± 0.15
TPCs ^2^ (mg GAE g^−1^)	73.36 ± 7.66	2.16 ± 0.13 ^B^	2.44 ± 0.11 ^A^
AOA ^2^ (µmol TEAC g^−1^)	496.12 ± 19.23	45.09 ± 2.15 ^B^	52.41 ± 3.11 ^A^

^1^ Data are reported as mean relative percentages of the total FAMEs ± SE. ^2^ Data are reported on a DM basis. TPCs = Total Phenolic Compounds, AOA = Antioxidant Activity. ^A,B^ Different letters indicate significant differences between the two groups (*p* < 0.05).

**Table 2:** Physical and chemical characterization of the raw *longissimus dorsi* muscle from lambs fed with a standard diet (CTR) and lambs fed with a diet containing grape pomace (GP+).

Physical Trait (%)	CTR	GP+	*p*
Drip loss (DL)	1.16 ± 0.50	1.08 ± 0.30	ns
Cooking loss (CL)	35.04 ± 2.11	33.93 ± 2.79	ns
Chemical composition (%)			
Dry matter (DM)	20.12 ± 2.04	21.16 ± 1.32	ns
Total lipids ^1^	10.56 ± 4.59	10.21 ± 4.51	ns

Data are reported as mean ± SD. ns = not significant. ^1^ Data are reported on a DM basis.

**Table 3:** Chromatic coordinates evaluated in raw meat collected 2 (T2) and 7 (T7) days *post mortem* from lambs fed with a standard diet (CTR) and lambs fed with a dietary grape pomace supplementation (GP+).

Coordinates	T2			T7		
	CTR	GP+	*p*	CTR	GP+	*p*
L*	43.4 ± 4.79	41.56 ± 3.37	ns	41.15 ± 4.50	39.71 ± 3.59	ns
a*	16.13 ± 1.39 ^A^	15.92 ± 2.61 ^A^	ns	8.53 ± 1.23 ^B^	7.77 ± 1.56 ^B^	ns
b*	5.34 ± 1.08	4.74 ± 1.49	ns	6.47 ± 1.06	6.36 ± 1.73	ns

Data are reported as mean ± SD. L* = lightness; a* = redness; b* = yellowness; ns = not significant. ^A,B^ Different letters indicate significant differences due to the storage time (*p* < 0.01).

**Table 4:** Fatty acid profiles in meat samples obtained from lambs fed a standard diet (CTR) and lambs that received the dietary supplementation with grape pomace (GP+).

Fatty Acid ^1^	CTR	GP+	*p*
C8:0	0.05 ± 0.01	0.04 ± 0.01	ns
C10:0	0.27 ± 0.09	0.21 ± 0.08	ns
C12:0	0.44 ± 0.12	0.38 ± 0.14	ns
C14:0	5.12 ± 0.83	4.16 ± 0.78	ns
C16:0	26.76 ± 2.73	24.61 ± 1.51	ns
C18:0	16.79 ± 2.19	19.54 ± 1.81	*
C20:0	0.14 ± 0.03	0.10 ± 0.02	ns
C22:0	0.46 ± 0.10	0.45 ± 0.12	ns
C24:0	0.43 ± 0.05	0.36 ± 0.12	ns
SFA	50.46 ± 4.59	49.85 ± 4.44	ns
C14:1 c9	0.66 ± 0.12	0.59 ± 0.10	ns
C16:1 c9	1.59 ± 0.18	1.45 ± 0.24	ns
C18:1 t11	1.44 ± 0.23	2.32 ± 0.23	**
C18:1 c9	28.72 ± 2.82	27.55 ± 3.19	ns
C18:1 c11	1.50 ± 0.23	1.36 ± 0.21	ns
MUFA	33.91 ± 3.11	33.27 ± 3.04	ns
C18:2 c9, c12	10.67 ± 0.93	11.97 ± 0.68	ns
C18:3 c9, c12, c15	0.67 ± 0.08	0.61 ± 0.06	ns
CLA	1.07 ± 0.09	1.22 ± 0.08	**
PUFA	12.41 ± 1.21	13.8 ± 1.28	ns
Others	3.22 ± 0.33	3.08 ± 0.31	ns
AI	17.08 ± 4.34	17.34 ± 4.67	ns
TI	9.36 ± 1.51	9.51 ± 1.51	ns

^1^ Data are reported as mean relative percentages (%) on total FAMEs ± SD; AI = Atherogenic index; TI = Thrombogenic Index; ns = not significant; * *p* <0.05; ** *p* < 0.01.

**Table 5:** Volatile compounds (VOCs) identified in *longissimus dorsi* samples obtained from lambs fed with a standard diet (CTR) and lambs fed a dietary supplementation with grape pomace (GP+). Samples were analyzed after cooking (T0) and after 5 days of storage (T5) at 4 °C.

	VOCs ^1^	T0			T5		
		CTR	GP+	*p*	CTR	GP+	*p*
Alcohols	1-Pentanol	1.63 ± 0.21 ^A^	1.43 ± 0.19 ^A^	ns	0.27 ± 0.04 ^B^	0.25 ± 0.03 ^B^	ns
	1-Octen-3-ol	5.38 ± 0.55 ^B^	5.87 ± 0.61 ^B^	ns	6.74 ± 0.65 ^B^	9.03 ± 0.88 ^A^	**
	2-Octen-1-ol, (Z)-	0.39 ± 0.05 ^B^	0.46 ± 0.06 ^B^	ns	1.14 ± 0.12 ^A^	0.99 ± 0.11 ^A^	ns
	1-Octanol	1.58 ± 0.19 ^A^	1.72 ± 0.22 ^A^	ns	0.76 ± 0.09 ^B^	0.67 ± 0.09 ^B^	ns
Aldehydes	Hexanal	71.47 ± 5.44 ^C^	70.08 ± 4.88 ^C^	ns	79.77 ± 2.03 ^A^	76.55 ± 1.65 ^B^	*
	Heptanal	1.32 ± 0.15	1.61 ± 0.17	ns	1.19 ± 0.13	1.38 ± 0.15	ns
	4-Pentenal, 2,2-dimethyl-	0.21 ± 0.08	0.12 ± 0.06	ns	nd	nd	ns
	Nonanal	4.98 ± 0.52 ^B^	4.25 ± 0.45 ^B^	ns	5.86 ± 0.46 ^B^	7.50 ± 0.67 ^A^	*
	Octanal	1.50 ± 0.18	1.77 ± 0.20	ns	1.46 ± 0.16	1.59 ± 0.18	ns
	2-Decenal, (E)-	0.39 ± 0.06	0.42 ± 0.06	ns	nd	nd	ns
Aromatic Hydrocarbons	Ethylbenzene	5.65 ± 0.59	6.19 ± 0.63	ns	5.08 ± 0.37	5.42 ± 0.41	ns
	O-xylene	4.21 ± 0.47	4.36 ± 0.49	ns	3.78 ± 0.39	4.58 ± 0.51	ns
	Benzene, 1,3-dimethyl-	1.57 ± 0.28	2.47 ± 0.31	ns	1.29 ± 0.16	1.37 ± 0.15	ns
	Benzene, 4-ethyl-1,2-dimethyl-	0.62 ± 0.11	0.69 ± 0.10	ns	1.24 ± 0.13	1.31 ± 0.15	ns
Ketones	2,3-Octanedione	8.07 ± 0.78 ^A^	8.06 ± 0.75 ^A^	ns	0.34 ± 0.05 ^B^	0.30 ± 0.04 ^B^	ns
Esters	Propanoic acid, 2-methyl-, butyl ester	1.59 ± 0.15	1.51 ± 0.17	ns	nd	nd	ns

^1^ Volatile Compounds (VOCs) are reported as mean relative percentages (%) on total detected VOCs ± SD. nd = not detectable; ns = not significant; * *p* < 0.05; ** *p* < 0.01. ^A,B,C^ Different letters indicate significant differences due both to the storage time and to the feeding treatment (*p* < 0.05).

## Data Availability

The data presented in this study are available on request from the corresponding author.
